# Do Anxiety Symptoms Mediate the Association Between Cannabis Use Frequency and Psychotic-Like Experiences in Emerging Adult Undergraduates?

**DOI:** 10.1177/07067437231176900

**Published:** 2023-05-17

**Authors:** Haley C. R. Bernusky, Philip G. Tibbo, Patricia J. Conrod, Fakir Md. Yunus, Matthew T. Keough, Kara D. Thompson, Marvin D. Krank, Allyson F. Hadwin, Sherry H. Stewart

**Affiliations:** 1 Department of Psychiatry, 3688 Dalhousie University, Halifax, Nova Scotia, Canada; 2 Département de psychiatrie et d’addictologie, 5622 Université de Montréal, Montréal, Québec, Canada; 3 Department of Psychology and Neuroscience, 3688 Dalhousie University, Halifax, Nova Scotia, Canada; 4 Department of Psychology, York University, North York, Ontario, Canada; 5 Department of Psychology, 1270 St. Francis Xavier University, Antigonish, Nova Scotia, Canada; 6 Department of Psychology, University of British Columbia, Kelowna, British Columbia, Canada; 7 Department of Educational Psychology and Leadership Studies, 8205 University of Victoria, Victoria, British Columbia, Canada

**Keywords:** anxiety, cannabis, psychotic-like experiences, mediation, biological sex, moderation, conditional process model, path analysis, emerging adults

## Abstract

**Objective:**

Cannabis is commonly used by Canadian emerging adults (ages 18–25 years), many of whom attend post-secondary institutions. Frequent cannabis use is linked with psychotic-like experiences (PLEs); however, the exact nature of this association remains unclear. Anxiety symptoms may mediate this association, as they are prevalent in emerging adults and have been independently linked with both cannabis use and PLEs. Past work found that anxiety mediated the association between cannabis use frequency and attenuated positive psychotic symptoms (further along the psychosis continuum than PLEs), however this research had yet to be validated in the Canadian population, and trait rather than state anxiety (frequency of anxiety symptoms) was studied. Thus, our primary objective was to examine if anxiety symptoms mediated the association between cannabis use frequency and PLEs in Canadian emerging adult undergraduates. Despite known sex differences in cannabis use, expression of anxiety, and PLEs, past work did not evaluate the potential impact of biological sex on the anxiety-mediated model, and thus is the secondary objective of the present study.

**Methods:**

1,266 first-/second-year emerging adult undergraduates from five Canadian universities provided cross-sectional, self-report survey data in fall 2021 semester. Validated measures of cannabis use frequency, anxiety, and PLEs were administered.

**Results:**

Path analyses supported mediation from cannabis use to PLEs through anxiety (*b*  =  0.07, *P* < 0.001, 95% bootstrap CI [0.03, 0.10]). No direct effect was found (*P*  =  0.457), suggesting that the cannabis-to-PLEs association was mediated by anxiety. Mediation did not depend on biological sex (i.e., bootstrapped 95% CIs crossed zero).

**Conclusions:**

Anxiety symptoms mediated the association between cannabis use and PLEs in emerging adults regardless of their biological sex. Assuming replication in prospective research, results highlight anxiety as an important intervention target in frequent cannabis-using emerging adults, to potentially prevent development/worsening of PLEs, and in turn psychotic illness.

## Abrégé

**Objectif:**

Le cannabis est communément utilisé par les jeunes adultes canadiens (âgés de 18 à 25 ans), dont bon nombre fréquentent les institutions post-secondaires. L’utilisation fréquente du cannabis est liée aux expériences de type psychotique (ETP); cependant, la nature exacte de cette association demeure imprécise. Les symptômes anxieux peuvent être un intermédiaire de cette association, car ils sont prévalents chez les nouveaux adultes et qu’ils sont indépendamment liés tant à l’utilisation du cannabis qu’aux ETP. Des travaux antérieurs ont constaté que l’anxiété exerce une médiation sur l’association entre la fréquence d’utilisation du cannabis et les symptômes psychotiques positifs atténués (plus éloignés sur le continuum de la psychose que les ETP). Toutefois, cette recherche restait encore à valider dans la population canadienne, et l’anxiété de trait plutôt que l’anxiété d’état (fréquence des symptômes anxieux) était étudiée. Notre objectif principal était donc d’examiner si les symptômes anxieux étaient médiateurs de l’association entre la fréquence d’utilisation du cannabis et les ETP chez les jeunes étudiants adultes de premier cycle. Malgré les différences connues entre les sexes concernant l’utilisation du cannabis, l’expression de l’anxiété et les ETP, la recherche antérieure n’a pas évalué l’effet potentiel du sexe biologique sur le modèle médié par l’anxiété, ce qui est donc l’objectif secondaire de la présente étude.

**Méthodes:**

1 266 nouveaux adultes de première ou deuxième année du premier cycle de 5 universités canadienne ont fourni des données d'enquête transversales auto-déclarées au semestre d’automne 2021. Des mesures validées de la fréquence d’utilisation du cannabis, de l’anxiété et des ETP ont été administrées.

**Résultats:**

Les analyses de trajectoire soutenaient la médiation entre l’utilisation du cannabis et les ETP par l’anxiété (b = , 07, p <, 001, IC bootstrap à 95% [,03 à 10]). Aucun effet direct n’a été trouvé (p = , 457), suggérant que l’association entre cannabis et ETP était médiée par l’anxiété. La médiation ne dépendait pas du sexe biologique (c.-à-d. les IC bootstrap à 95% ne croisaient pas zéro).

**Conclusions:**

Les symptômes anxieux étaient un médiateur de l’association entre l’utilisation du cannabis et les ETP chez les jeunes adultes sans égard à leur sexe biologique. En supposant la réplication dans la recherche prospective, les résultats soulignent l’anxiété comme étant une importante cible d’intervention chez les jeunes fréquents utilisateurs de cannabis, afin de prévenir potentiellement le développement ou l’aggravation des ETP, puis de la maladie psychotique.

Many Canadian emerging adults (aged 18–25) use cannabis. In 2021, ∼49% of those aged 20–24 years reported cannabis use;^1^ 26% of male and 24% of female emerging adult cannabis users reported daily/almost daily use.^1^ Of Canadian emerging adults attending post-secondary institutions pre-COVID-19 pandemic, 48% reported past-year cannabis use; 9% of all male and 6% of all female students reported past month daily/almost daily use.^2^

Research shows an association between cannabis use and psychosis, with higher rates of use observed in patients with, versus without, psychosis.^3^ This association is dose-dependent, with higher frequency and THC potency of cannabis use significantly increasing the risk for psychosis.^3–5^ Daily cannabis users are  > three times more likely to develop a primary psychotic disorder compared to never users, increasing to nearly five-times the risk with higher potency cannabis.^5^ Cannabis risk mitigation strategies may benefit from examining processes earlier in psychotic illness development, specifically psychotic-like experiences (PLEs).

PLEs are defined as non-persistent changes in thoughts, perceptions, and behaviors that do not impede overall functioning (e.g., perceptual abnormalities, magical thinking), thus not qualifying as primary psychotic disorder symptoms.^6,7^ According to the psychosis continuum hypothesis,^7^ the same factors that increase risk for psychotic disorders (e.g., frequent cannabis use) underlie the entire spectrum, thus also contributing to the prevalence of PLEs in the general population.^6,8,9^ Individuals with PLEs are at risk of developing psychotic disorders.^10,11^ As first episodes of psychosis tend to present in emerging adulthood,^12^ examining links between cannabis use and symptoms earlier along the psychosis continuum is important given the high prevalence of cannabis use in this cohort. Thus, it is important to expand our limited understanding of the link between cannabis use and the development of PLEs, including underlying mechanisms.

Anxiety is common in Canadian emerging adults: approximately 23% of those aged 18–24 reported moderate-to-severe anxiety,^13^ which typically increases in the first year of post-secondary education.^14^ Frequent cannabis use is a risk factor for maintaining/worsening anxiety symptoms in adolescents and emerging adults. Early onset users, those using weekly or more, and those with greater lifetime cannabis exposure were all more likely to report elevated anxiety symptoms at later assessments compared to less frequent users.^14–17^ Anxiety is also a risk factor for PLEs: emerging adults with anxiety disorders are approximately five times more likely to score in the highest (versus lowest) quartile of PLE scores compared to non-anxious peers.^10,18^ Given these established links, anxiety appears an important potential mediating variable in clarifying the cannabis use-to-PLEs association in emerging adults.

Reeves and colleagues demonstrated that the link between cannabis use frequency and attenuated positive psychotic symptoms (APPS) was mediated by trait anxiety in an American sample aged 17–35 years.^19^ Their results require replication and extension for four reasons. First, their data were collected in 2013 from a US sample in a state where recreational cannabis use remains illegal,^20^ potentially limiting generalizability to the current Canadian undergraduate context. In Canada, recreational cannabis use was legalized in 2018,^1^ increasing accessibility/normalization of cannabis, and potentially increasing participants’ willingness to report actual cannabis use levels.^21,22^ Second, Reeves et al.^19^ measured trait anxiety (a stable personality trait)^23^ which is less conceptually relevant as a mediator of the cannabis use-to-PLEs association than anxiety symptom frequency. Their results warrant replication using a validated measure of anxiety symptoms, which would more accurately assess if higher frequency cannabis use is a risk factor for increased frequency of anxiety symptoms,^14–17^ which, in turn, increases the risk for PLEs.^10,18^ Third, Reeves et al. specified their outcome as APPS^19,24^ which are further along on the psychosis continuum^7^ (i.e., closer to psychotic disorder) than PLEs. Keeping earlier risk mitigation for psychotic disorders in mind, it is important to examine if anxiety-mediation can be demonstrated earlier along the psychosis spectrum.^6–9^

Lastly, Reeves et al.^19^ did not evaluate the potential moderating impact of sex on the anxiety mediation model. Males use cannabis more frequently and in higher quantities, are more likely to have a cannabis use disorder, and are at higher risk for primary psychotic disorders than females.^16,25,26^ Comparatively, females are at greater risk for developing anxiety disorders than males.^14,27^ Moreover, female versus male daily cannabis users have greater than five times the risk of developing anxiety.^16,27^ Anxiety mediation of the cannabis use-PLEs link suggested by Reeves et al.^19^ may also differ by sex.

**Figure 1.**
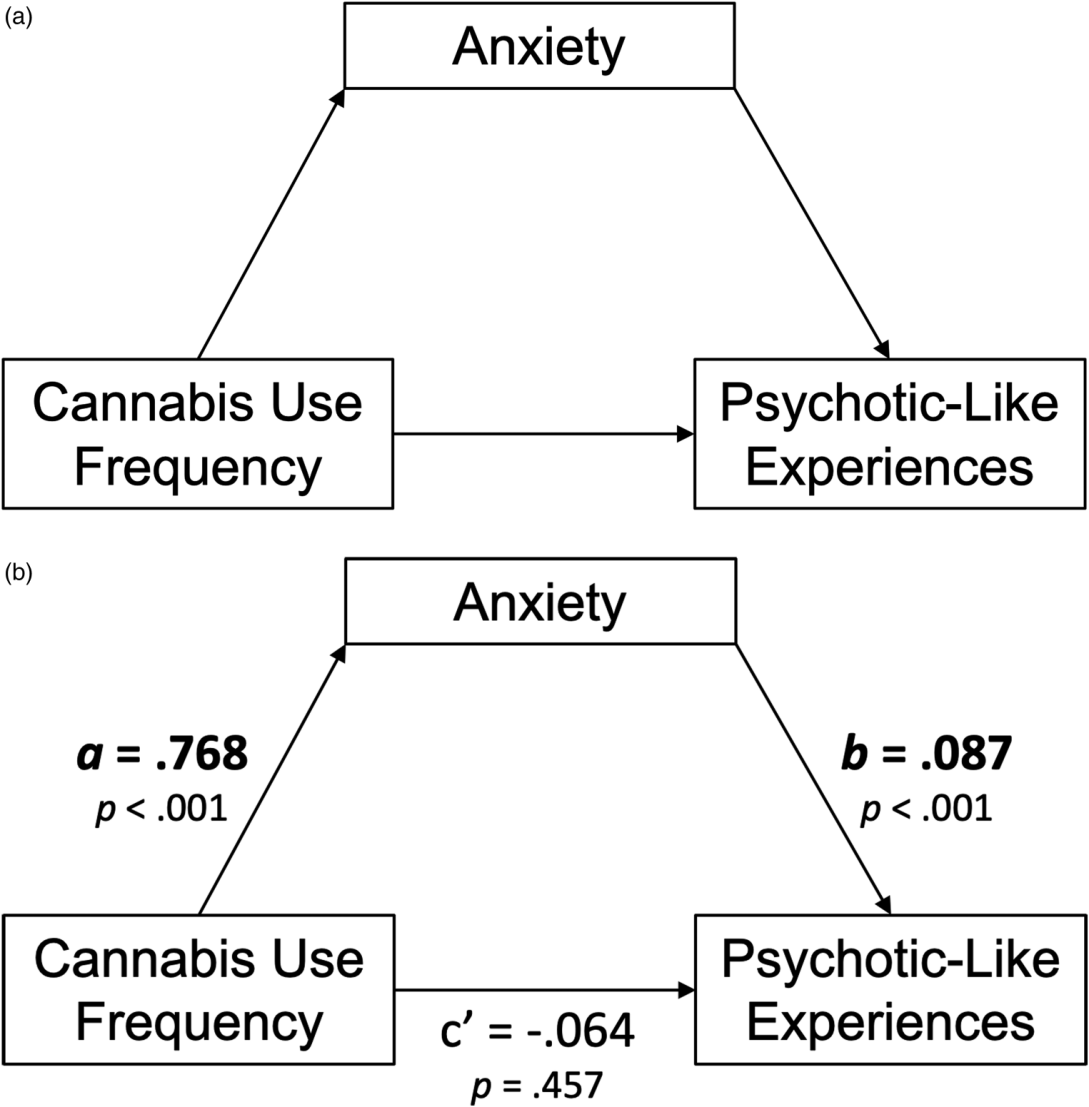
(a) Conceptual model of the simple mediation model. (b) *N*  =  1,251. Path diagram of the simple mediation model.

**Figure 2.**
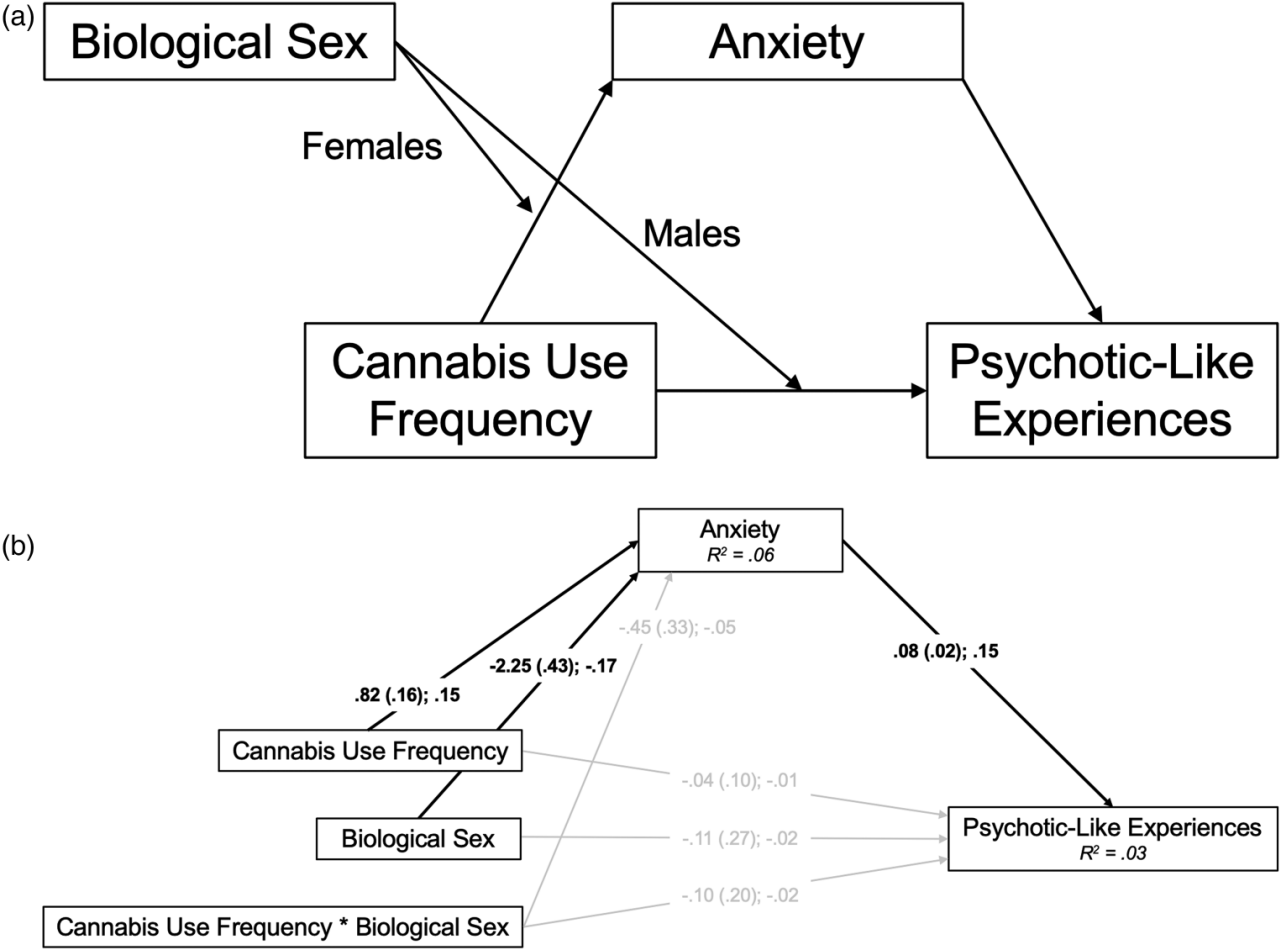
(a) Conceptual model of the conditional process (moderated mediation) model. (b) *N*  =  1,248. Path diagram of the conditional process (moderated mediation) model; females  =  0, males  =  1. Path estimates presented as unstandardized coefficient (standard error); standardized coefficient. Bold denotes specified paths that were statistically significant (*P* < 0.05). *R^2^* values represent the proportion of variance in the dependent variable that can be explained by the independent variables, significant at *P* < 0.001. MLR estimation was used to account for skewness.

Considering these gaps, we aimed to replicate previous mediational findings^19^ in a sample of Canadian emerging adult undergraduates. We also attempted to extend Reeves et al.'s^19^ model by specifying anxiety symptoms as the mediator and PLEs as the outcome, and by testing potential sex moderation. We hypothesized that (H1) more frequent cannabis use would be indirectly associated with greater PLEs through greater anxiety symptoms (Figure 1a). We hypothesized that sex would moderate this mediational model, with (H2) the cannabis-to-anxiety symptoms path (i.e., the *a*-path in the indirect effect) proving statistically stronger for females and (H3) the direct path from cannabis use frequency to PLEs proving stronger for males (Figure 2a).

## Materials & Methods

### Participants and Procedures

Hypotheses were tested using cross-sectional, self-report survey data collected in fall 2021 from first-/second-year emerging adult undergraduates aged 18–25 years. Students were recruited from five Canadian universities to participate in the UniVenture substance misuse prevention project^1^. All sites received research ethics board approval to conduct the survey. Recruitment occurred via online undergraduate research participation pools, campus-/faculty-wide electronic communications, social media/website advertisements (e.g., Facebook, Instagram), and/or on-campus posters/information booths. Participants gave written, electronic informed consent before completing online surveys. Participants received partial credit for an eligible psychology course or a gift card as compensation for their participation.

### Measures

**Demographics.** An author-compiled demographic measure identified emerging adult students (between 18 and 25 years)^31^ and provided data on biological sex assigned at birth (male/female)^2^ for the conditional process analysis. Site information was obtained to control for site effects.

**Cannabis Use Frequency.** The 20-item Co-Venture Drug Use Battery,^32^ a shortened version of the psychometrically-sound Detection of Alcohol and Drug Problems in Adolescents (DEP-ADO) questionnaire,^33,34^ measured participants’ frequency of use of several substances including cannabis (the predictor). Alcohol and tobacco use was obtained to control for co-morbid substance use (common in cannabis users,^1,2^ posing additional risk for PLEs).^3,4^ Participants responded using a 10-point frequency scale; the option *I prefer not to say* was treated as missing. For scoring, response options were recoded to match those of the first item of the AUDIT-C:^35^ 0  =  *Never*, 1  =  *Monthly or less*, 2  =  *2–4 times a month,* 3  =  *2–3 times a week*, 4  =  *4*  *+*  *times a week*. Those indicating daily/almost daily cannabis use were considered at highest risk for problematic cannabis use, including cannabis use disorder.^27^

**Anxiety.** The seven-item Generalized Anxiety Disorder scale (GAD-7)^36^ measured participants’ past-three-month frequency of anxiety symptoms, where higher scores reflected more frequent anxiety symptoms. Response options included: 0  =  *Not at all*, 1  =  *Several days*, 2  =  *More than half the days*, 3  =  *Nearly every day*, or *I prefer not to say* (treated as missing). Scores of 10 and 15 (out of a possible 21) are cut-offs signaling potentially clinically significant moderate and severe levels of anxiety, respectively.^36^ This study yielded Cronbach's α  =  0.92 for the GAD-7, consistent with previous work.^36^ Total GAD-7 scores served as the mediator in our models.

**Psychotic-Like Experiences.** The Psychotic-Like Experiences Questionnaire (PLEQ)^37,38^ is a validated measure of PLEs over the past three months in youth; higher scores reflect more severe symptoms. Five items predictive of schizophreniform disorder^11^ were adapted from the Diagnostic Interview Schedule for Children^39^ (e.g., “Have you ever heard voices that other people could not hear?”). Four additional items assessed a broader range of PLEs^37,38^ (e.g., “Do you have any special powers that other people don’t have?”). Options included: 0  =  *Not true*, 1  =  *Somewhat true*, 2  =  *Certainly true*, or *I prefer not to say* (treated as missing) for total scores from 0 to 18.^37,38^ Total scores of four or more indicate the presence of PLEs.^40^ This study yielded Cronbach's α  =  0.78 for the PLEQ, consistent with previous work.^38,41^ Total PLEQ scores served as the outcome in our models.

### Statistical Analysis

R version 4.2^42^ and SPSS Statistics version 28^43^ were used to clean the data/obtain descriptive statistics. Two path models tested our hypotheses: in the simple mediation model, cannabis use frequency was specified as a predictor of PLEs (outcome) through anxiety (mediator); in the conditional process model, sex was examined as a moderator of the indirect (through anxiety) and direct pathways from cannabis use to PLEs. Here, cannabis use, sex, and the cannabis use by sex interaction term were specified as predictors of anxiety and of PLEs. Bias-corrected bootstrapped 95% confidence intervals (CI) were used to determine the presence and magnitude of indirect effects. Mplus version 8.7^44^ was used to conduct the path analyses with FIML for missing data, and a maximum likelihood estimator robust to non-normal distributions (MLR), accounting for skewness. We considered both models supported if the relevant 95% CIs did not include zero.^45^

**Sensitivity Analyses.** Three additional models determined if any predicted effects supported in the main analyses remained significant after controlling for plausible confounds (i.e., study site, alcohol/tobacco use frequency).

**Power and Sample Size.** The 20-case-per-parameter rule of thumb^46^ suggests that *N*  =  180 participants would be needed to achieve 80% power. Thus, our much larger sample (*N*  =  1,266) was sufficiently powered to detect sex moderation of the direct/indirect effects.

## Results

### Demographic Variables

Cross-site data were merged, scored, and cleaned: 1,875 participants were excluded, having withdrawn from participation (*n*  =  1,218, i.e., closed browser prior to completion), ineligibility (*n*  =  504, e.g., not 18–25 years), and unreliable responding (*n*  =  153, e.g., inconsistent responses to similar items), resulting in a final sample of 1,266 participants (see Table 1 for demographics).

**Table 1.** Descriptive Statistics–Demographic Variables.

Demographic variable	Overall sample (*N* = 1,266)
Age (years), *M* (*SD*); [range]	19.13 (1.49); [18–25]
Sex assigned at birth, *n* (%)	Female = 849 (67.1%)
	Male = 414 (32.7%)
	Missing = 3 (0.2%)
Gender identity, *n* (%)	Woman = 825 (65.2%)
	Man = 409 (32.3%)
	Transgender = 3 (0.2%)
	Non-binary = 21 (1.7%)
	Two-spirit = 1 (0.1%)
	Other = 6 (0.5%)
	Missing = 1 (0.1%)
Year of study, *n* (%)	First-year undergraduate = 717 (56.6%)
International student status, *n* (%)	Yes = 170 (13.4%)
	No = 1,095 (86.5%)
	Missing = 1 (0.1%)
Visible minority status, *n* (%)	Yes = 337 (26.6%)
	No = 877 (69.3%)
	Missing = 51 (4.1%)
Parental status, *n* (%)	Yes, I have children = 3 (0.2%)
Employment status, *n* (%)	Full-time = 4 (0.3%)
	Part-time = 126 (10.0%)
	Unemployed = 455 (35.9%)
	I prefer not to say = 654 (51.7%)
	Missing = 27 (2.1%)

*Note. M*  =  mean; *SD*  =  standard deviation.

### Clinical Variables

Table 2 provides the means and bivariate correlations of the clinically relevant variables under study and possible confounding variables. We found statistically significant positive correlations between cannabis use frequency and anxiety, and between anxiety and PLEs, the two paths constituting the hypothesized indirect effect.

**Table 2.** Descriptive Statistics and Bivariate Correlations—Clinical Variables.

Variable	*M*	*SD*	Skew	Kurt	1.	2.	3.	4.
1. Cannabis use frequency	0.87	1.15	1.48	1.40				
2. Psychotic-like experiences	3.54	3.29	1.33	1.90	–0.001			
3. Anxiety	10.26	6.06	0.19	−1.08	0.145**	0.157**		
4. Alcohol use frequency	1.44	1.07	0.34	−0.82	0.501**	–0.046	0.071*	
5. Tobacco use frequency	0.75	1.24	1.78	1.95	0.551**	0.083**	0.123**	0.466**

*Note. M*  =  mean; *SD*  =  standard deviation; Skew  =  skewness; Kurt  =  kurtosis.

* Correlation significant at .05 level; ** Correlation significant at .01 level (2-tailed).

**Cannabis Use Frequency.** Half (50.6%) of the sample reported lifetime cannabis use (48.6% never use, 0.8% missing). Of the total sample, 33.2% reported past-three-month use, and 6.5% daily/almost daily use. On average, participants used cannabis just less than once per month (Table 1). Females (*M*  =  0.92 times per month, *SD*  =  1.19) used cannabis significantly more frequently than males (*M*  =  0.77 times per month, *SD*  =  1.04), *t*(914.56)  =  2.29, *P*  =  0.022. Furthermore, cannabis use frequency differed significantly by site (S2 > all others, *P* < 0.001).

**Anxiety.** Of the total sample, 49.6% reported moderate, and 27.9% reported severe, anxiety over the past three months^36^ (Table 1). Females (*M*  =  11.14, *SD*  =  6.04) were significantly more anxious than males (*M*  =  8.41, *SD*  =  5.69), *t*(844.10)  =  7.77, *P* < 0.001. Furthermore, anxiety levels differed significantly by site (S2 > S4, S5; S1 > S4, *P* < 0.05).

**Psychotic-Like Experiences.** Three-quarters (78.6%) of our sample reported any endorsement of PLEs in the past three months; 37.0% scored four or more, indicating the presence of PLEs.^40^ On average, participants reported total PLEQ scores of 3.54/18^37,38^ (Table 1), doubling the average total PLEQ score reported by emerging adults in a recent (pre-pandemic) cohort study (1.64/18 in the past year).^40^ Males (*M*  =  3.29, *SD*  =  3.28) and females (*M*  =  3.65, *SD*  =  3.26) did not differ significantly in PLEs (*P*  =  0.073), but there was significant variation by site (S3 > all others, *P* < 0.01).

### Simple Mediation Model

This model tested H1 that more frequent cannabis use would be associated with greater PLEs through greater anxiety symptoms. Both *a-* and *b-*paths constituting the indirect (mediated) effect were statistically significant at *P* < 0.001, as was the associated indirect effect (*b*  =  0.07, *P* < 0.001, 95% CI [0.03, 0.10]); thus, we found support for H1. After accounting for the indirect effect, there was no evidence of a remaining direct effect of cannabis use frequency on PLEs (*c’*  =  −0.06, *P*  =  0.457), suggesting anxiety symptoms fully mediate the cannabis-to-PLEs association in emerging adult undergraduates (see Figure 1b for full path diagram). The direction, magnitude, and significance of effects remained when controlling for study site and co-morbid alcohol and tobacco use.

### Conditional Process Model

This model examined sex moderation of the simple mediation model (H2 and H3). The cannabis use frequency by sex interaction term did not predict anxiety symptoms or PLEs; thus, the anxiety mediation model did not depend on sex. Consistent with this lack of sex moderation effects, the 95% CIs crossed zero for both the indirect effect from the interaction term to PLEs through anxiety symptoms (95% CI  =  −0.093, 0.018], and the direct effect of the interaction term to PLEs [95% CI  =  −0.493, 0.303]. Inconsistent with H2 and H3, sex did not moderate the direct or indirect effects of cannabis use on PLEs (Figure 2b).^3^^4^

## Discussion

Our primary objective was to examine whether anxiety symptoms mediated the association between cannabis use frequency and PLEs. Results supported anxiety mediation of the link between cannabis use frequency and PLEs, consistent with H1. Our secondary objective was to examine the potential moderating effect of sex on the hypothesized anxiety-mediated model. Contrary to our hypotheses, there was no evidence of sex moderation.

Reeves et al.^19^ reported that the cannabis use-APPS association was mediated by trait anxiety in a US sample aged 17–35 years. We successfully replicated this anxiety-mediated model in the Canadian context (where cannabis is currently legal/widely available)^1^, and in a sample focused specifically on the developmentally vulnerable period of emerging adulthood (ages 18–25)^31^ when cannabis use is common^1^ and psychosis is most likely to onset.^12^ Moreover, results supported anxiety mediation despite changing both mediator and outcome variables relative to Reeves and colleagues^19^: mediation was supported when the mediator was current (past-three-month) anxiety symptoms, measured by the GAD-7,^36^ rather than trait anxiety.^19^ Conceptually, anxiety symptoms are more state-like (variable over time) than trait anxiety (a stable personality trait)^23^ and thus, a more relevant mediator in the cannabis use-PLE association. We also successfully extended Reeves et al.'s anxiety mediation model using PLEs as the outcome (versus APPS).^19,24^ PLEs are less severe/earlier along the psychosis continuum,^7^ increasing the relevance of results to early identification and potential prevention of psychosis development in vulnerable emerging adults. This supported mediation model provides further evidence that frequent cannabis use and, in turn, heightened anxiety, are important potential risk factors for PLEs to target in interventions.

We extended Reeves et al.^19^ by testing the potential sex-moderation effects on the anxiety-mediation model. Results did not support our sex-moderation hypotheses, suggesting that anxiety mediates the trajectory from frequent cannabis use to PLEs in emerging adults regardless of sex.

Historically, research has supported that males tended to use cannabis more frequently and in higher quantities compared to females.^1,2,16,25,26^ However, national cannabis surveys show that over time, the gap between sexes in cannabis use has narrowed, with rates of use in females rising to match that of males, a phenomenon known as convergence.^47,48^ In the present sample, females used cannabis significantly more frequently than males. This might be attributed to the growing social acceptability of cannabis use in young females in Canada.^47^ The COVID-19 pandemic may have influenced results: 40% of Canadian emerging adults reported increasing their quantity, and 38% their frequency, of cannabis use during the pandemic.^1^ Common reasons for increasing use were increased stress, anxiety, and isolation/loneliness^1^; these predictors of greater cannabis use^17,49^ were particularly elevated among Canadian female versus male undergraduates during the pandemic.^50,51^

While anxiety symptoms were found to be moderately severe overall, females reported significantly more anxiety on the GAD-7 than males. This finding is consistent with literature showing that females are more anxious than males,^14,27^ and with reports that levels of anxiety have been significantly higher among females throughout the pandemic.^50,51^ Furthermore, PLEs were prevalent in this sample with students reporting an average of 3.54/18 on the PLEQ,^37,38^ nearly doubling previous reports (1.64/18 in the past year).^40^ ∼37% of the present sample scored four or more on the PLEQ, indicating the presence of PLEs.^40^ This rate is elevated compared to the 11.9% of a pre-pandemic sample of emerging adults who scored four or more found previously.^40^ As substance use, stress, anxiety, and isolation/loneliness increased during the pandemic,^1^ the elevation observed in PLE scores may also be explained by the present sample being taken during the pandemic. Indeed, a recent study compared inpatient psychiatric admissions during the COVID-19 pandemic to an earlier comparator period and found significantly more admissions related to substance use during the pandemic than before (45% vs. 28%).^52^ Contrasting literature showing males to be at higher risk for PLEs/primary psychotic disorders compared to females,^16,25,26^ the number of PLEs reported herein did not vary by sex. Equivalent reports of PLEs by males and females in the present study may be due to females in our sample using cannabis significantly more frequently than the males, given the known links between cannabis use and symptoms along the psychosis continuum.^3,6,7,9^

### Limitations

Firstly, mediation is considered a causal process that unfolds over time,^45^ however this study used cross-sectional data limiting causal inference.^53^ For example, this study assumed that anxiety symptoms preceded PLEs, however, it is possible that the experience of PLEs causes anxiety symptoms.^10^ For this reason, some argue against testing mediation with cross-sectional data.^54^ Others suggest that cross-sectional studies are reasonable when evaluating newer mediational models while keeping directional inference limitations in mind.^45,53^ Our results corroborate those found in a different sample using different measures,^19^ supporting this study as a useful early step in increasing our understanding of anxiety's role in the link between cannabis use and psychosis-related outcomes.

Secondly, data were collected via online self-report measures which may be prone to biased responding. Furthermore, the Co-Venture Drug Use Battery^32^ is a shortened version of the psychometrically-sound DEP-ADO,^33,34^ yet the reliability/validity of some specific individual items (e.g., cannabis use frequency) are unavailable. Possible mitigating factors of these limitations include that self-reported substance use has been shown to be acceptably accurate when confidentiality is assured and there are no risks of adverse consequences for responding truthfully (both true for this study).^55^ Furthermore, online questionnaires have been shown to reliably capture emerging adults’ self-reported substance use.^22^ Future work would benefit from clinician-administered interviews or biological drug testing, for example, to support self-report measures.

This study did not include certain clinically relevant variables such as participants’ family history of substance misuse, psychosis, and trauma.^3^ Future studies would benefit from inclusion and control of such variables, to allow a more nuanced interpretation of results.

Lastly, while our sensitivity analyses controlled for site effects and the substances most commonly co-used by cannabis users^1,2^ and those at clinical high-risk for psychosis^56^ other potential confounding variables may have impacted the study. For example, age of onset of cannabis use and other psychiatric comorbidities should be assessed and controlled for in future studies testing anxiety mediation.^3,10,16^

## Conclusion

The high frequency of cannabis use and high prevalence of moderate/severe anxiety and PLEs in this Canadian emerging adult undergraduate sample further supports the need to increase our understanding of the relationship between these variables. Our study moved toward clarifying one potential mechanism (anxiety symptoms) driving the link between cannabis use and PLEs, including assessing for sex moderation. Our results suggest the following possible clinical implications: that reducing anxiety symptoms in frequent cannabis-using emerging adults may help prevent the development/worsening of PLEs, and that anxiety symptoms as an intervention target may be relevant for psychosis risk-reduction among undergraduates regardless of sex.

## Notes

1. UniVenture study sites are located across Canada in various rural, small, and large urban centres, labelled Sites 1-5 to preserve their privacy per an agreement between sites participating in the UniVenture Partnership.^28^ Baseline UniVenture survey data analyzed in this study were collected during the COVID-19 pandemic (i.e., between September and November 2021). Infection rates during this time were highest in the community surrounding Site 5 (Quebec; 4,715.53 cases per 100,000 people), then Site 3 (Ontario; 3,942.67), Site 4 (British Columbia; 3,594.60), and lowest surrounding Sites 1 and 2 (Nova Scotia; 674.49).^29,30^ Site differences in the number of cases and associated differences in public health restrictions may have differentially impacted the data collected, prompting sensitivity analyses controlling for site as described in the main body of the paper (site-specific data not shown).

2. Biological sex and gender identity were largely congruent in the present sample (97.4%), thus precluding reliable analysis of subsamples beyond the gender binary (men, women). Our analyses focused on moderation by biological sex, promoting inclusivity by being able to retain data from those who identify differently.

3. Although not originally hypothesized, an anonymous reviewer asked us to examine the possibility of sex moderation on all paths in the model, specifically including the *b*-path from anxiety to PLEs. When we tested the fully moderated model, we found the same pattern of results: biological sex did not moderate any of the paths in the conditional process model, suggesting that anxiety is a relevant mediator of the link between cannabis use frequency and PLEs in Canadian emerging adult undergraduates regardless of their biological sex assigned at birth. We opted to retain the original, more parsimonious model described in the main text.

4. Although not originally hypothesized, an anonymous reviewer asked us to investigate the possibility of gender as a moderator of the simple anxiety-mediated model. To do so, we dichotomized our gender variable, retaining only those who indicated “man” or “woman” as their gender identity since the sample size of non-binary participants was too small to permit reliable analyses. We ran the model with dichotomous gender specified as the moderator, and the same pattern of results were obtained—the mediation model did not depend on gender identity (binary: men vs. women), suggesting that anxiety is a relevant mediator explaining the link between cannabis use frequency and PLEs for both those who identify as men and those who identify as women. We have not included these analyses in the main text but are available upon request.
